# Aortic Valve Sclerosis in High-Risk Coronary Artery Disease Patients

**DOI:** 10.3389/fcvm.2021.711899

**Published:** 2021-07-27

**Authors:** Veronika A. Myasoedova, Stefano Genovese, Laura Cavallotti, Alice Bonomi, Mattia Chiesa, Jeness Campodonico, Maurizio Rondinelli, Nicola Cosentino, Damiano Baldassarre, Fabrizio Veglia, Mauro Pepi, Francesco Alamanni, Gualtiero I. Colombo, Giancarlo Marenzi, Paolo Poggio

**Affiliations:** Centro Cardiologico Monzino Istituto di Ricovero e Cura a Carattere Scientifico (IRCCS), Milan, Italy

**Keywords:** aortic valve sclerosis, high-risk coronary artery disease, acute myocardial infarction, all-cause mortality, coronary artery bypass graft

## Abstract

**Background:** Current knowledge regarding the relationship between aortic valve sclerosis (AVSc), cardiovascular risk factors, and mortality in patients with known coronary artery disease (CAD) is still unclear. The present study aimed at investigating the prevalence of AVSc as well as its association with long-term all-cause mortality in high-risk CAD patients that has never been explored in large cohorts thus far.

**Methods and Results:** In this retrospective and observational cohort study we enrolled high-risk CAD patients, hospitalized at Centro Cardiologico Monzino (CCM), Milan, Italy, between January 2006 and December 2016. The morphology and function of the aortic valve were assessed from the recorded echocardiographic images to evaluate the presence of AVSc, defined as a non-uniform thickening of the aortic leaflets with no consequences on hemodynamics. Data on 5-year all-cause mortality was retrieved from a Regional database. Of the 5,489 patients initially screened, 4,938 (mean age 67 ± 11 years, 3,954 [80%] men) were enrolled in the study. In the overall population, AVSc was detected in 2,138 (43%) patients. Multivariable LASSO regression revealed that age, female gender, diabetes mellitus, previous MI, and left ventricular ejection fraction were independently associated with AVSc. All-cause mortality (adjusted hazard ratio: 1.29, 95%CI: 1.05–1.58) was significantly higher in AVSc than in non-AVSc patients.

**Conclusions:** AVSc is frequently detected in high-risk CAD patients and is associated with long-term mortality. Our findings corroborate the hypothesis that AVSc is an underestimated marker of systemic cardiovascular risk. Thus, AVSc detection may be used to improve long-term risk stratification of high-risk CAD patients.

## Introduction

Aortic valve sclerosis (AVSc) is an echocardiography finding characterized by non-uniform thickening of the aortic leaflets with no consequences on the left ventricular outflow tract (1). It is particularly common in the elderly (2) with studies reporting an estimated prevalence of 25–30% in patients over 65 years and up to 40% in those over 75 years (3–5). Epidemiological studies suggest that AVSc is associated with both all-cause and cardiovascular mortality, independently of age (5–7). AVSc shares many underlying mechanisms involved in atherosclerosis, including lipid deposition, oxidative stress, inflammation, and calcification (8). Besides, the risk factors associated with coronary artery disease (CAD), such as age, hypertension, dyslipidemia, and diabetes mellitus have been also associated with AVSc (1). Thus, it is not surprising that they often coexist in the same subject, with CAD patients having a high prevalence of AVSc (5). Nevertheless, AVSc has unique features, such as calcium predominance and slower progression compared to atherosclerosis (9, 10).

It has been hypothesized that AVSc, being the consequence of a spectrum of diseases, may have clinical importance and serve as a cardiovascular risk marker in patients without overt atherosclerosis (2). Indeed, it has been suggested that AVSc presence could underline a worse clinical condition, and thus, it might prompt clinicians toward more aggressive preventive strategies (1, 11). However, the current knowledge regarding the relationship between AVSc, cardiovascular risk factors, and mortality in patients with known CAD is incomplete. In particular, the prognostic relevance of AVSc in high-risk patients with CAD, such as those with acute myocardial infarction (AMI) or with multi-vessel CAD, has never been investigated in large cohorts.

The present study aimed to investigate the prevalence of AVSc and its association with cardiovascular risk factors, as well as with long-term all-cause mortality in a large cohort of high-risk patients with CAD.

## Methods

### Study Population

This is a retrospective, observational cohort study. We enrolled consecutive patients, hospitalized at Centro Cardiologico Monzino (CCM), Milan, Italy, between January 2006 and December 2016. Patients with multi-vessel disease undergoing coronary artery bypass graft (CABG) surgery and those with AMI (both ST- and non-ST-elevation myocardial infarction) were included in the study. Patients with significant valvular pathologies (e.g., aortic stenosis, mitral regurgitation, or rheumatic heart disease), major concomitant systemic conditions, such as malignancies, or poor echocardiographic images were excluded from the study. The study was approved by the Institutional Review Board and by the Ethical Committee of CCM (R553/17-CCM 591). It was possible to involve patients or the public in the design and conduct of our research. The investigation conformed to the principle outlined in the Declaration of Helsinki (1964).

### Study Protocol

All patients underwent a two-dimensional echocardiographic evaluation during the index event and demographic and clinical characteristics were collected at baseline. The 5-year all-cause mortality of these patients was retrieved from the Lombardy Regional registry. The first objective was to assess the prevalence of AVSc and the associations with cardiovascular risk factors in this specific high-risk clinical setting. The second purpose of the study was to evaluate AVSc association with 5-year all-cause mortality.

### Echocardiographic Evaluation

Experienced cardiologists of CCM performed the echocardiographic scans according to guidelines (12–14). The morphology and function of the aortic valve were assessed from the recorded echocardiographic images to evaluate the presence of AVSc, expressed as a dichotomous variable (yes or no). In case that the echocardiographic analysis of the aortic valve was not detailed, we evaluated the echocardiographic images before patients' discharge. The AVSc was identified according to criteria described by Gharacholou et al. (1), i.e., irregular, non-uniform thickening of portions of the aortic valve leaflets or commissures, or both; thickened portions of the aortic valve with an appearance suggesting calcification (i.e., bright echoes); non-restricted or minimally restricted opening of the aortic cusps; and peak continuous wave Doppler velocity across the valve <2 m/s. Moreover, we retrieved left ventricular ejection fraction (LVEF) measured at baseline in all enrolled patients.

### Statistical Analysis

Continuous variables are presented as mean ± SD. Categorical data are reported as frequencies and percentages. Group comparisons for continuous and categorical variables were performed by Student *t*-test for independent samples and by chi-square (χ^2^) test, respectively.

The association between the following variables and AVSc was assessed: cardiovascular risk factors (i.e., age, sex, body mass index [BMI], hypertension, dyslipidemia, diabetes mellitus, smoking habit, and previous AMI), LVEF, and estimated glomerular filtration rate (eGFR). To estimate the distribution of each standardized beta regression coefficient and, therefore, assess the strength of the association between the dependent (AVSc) and the independent variables, we implemented a strategy that combined the bootstrap resampling procedure and the logistic or Cox Least Absolute Shrinkage and Selection Operator (LASSO) regression (15), when appropriate. *P*-values were computed counting the number of times a beta coefficient is different from zero (which means no association) out of the total number of bootstrap iterations (1,000).

Kaplan-Meier analysis was used to generate time-to-event curves for 5-year mortality in patients with and without AVSc and the log-rank test was used to compare strata. Multivariable Cox regression analysis was implemented to take into account confounding effects, according to the significant variables in the LASSO regression analyses. The net reclassification improvement (NRI) and the integrated discrimination improvement (IDI) were performed, exploiting the “survIDINRI” R package (https://cran.r-project.org/web/packages/survIDINRI/index.html).

Differences were deemed significant if the *p*-value was < 0.05. Analyses were performed by R (v. 4.0.0) (https://www.R-project.org/) and SPSS statistical software 26.

## Results

**Figure 1:**
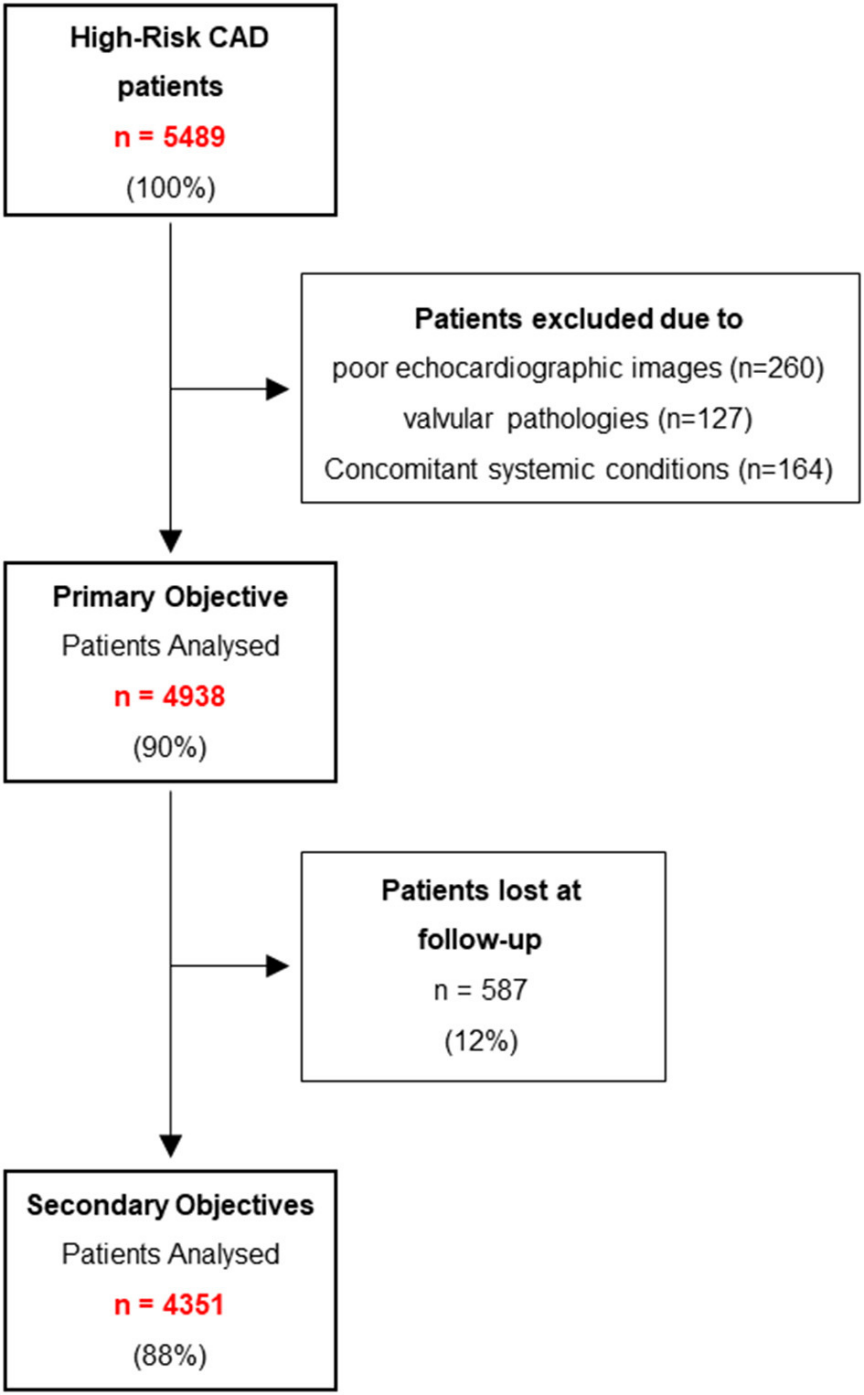
Flow diagram of the study. CAD, coronary artery disease.

Of the 5,489 patients initially screened, 4,938 (mean age 67 ± 11 years, 3,954 [80%] men) were enrolled in the study (Figure 1). The demographic and clinical characteristics of the final study population, as well as of patients with and without AVSc are shown in Table 1, while the characteristics of patients lost at follow-up are shown in Supplementary Table 1.

**Table 1:** Demographic and clinical characteristics of the high-risk coronary artery disease patients divided by the presence of aortic valve sclerosis.

**Variables**	**All (*n* = 4,938)**	**AVSc (*n* = 2,138)**	**No AVSc (*n* = 2,800)**	***p*-value**
Age, years	66.8 ± 10.7	71.1 ± 9.6	63.6 ± 10.4	** <0.0001**
Male *n*, (%)	3,954 (80)	1,619 (76)	2,335 (83)	** <0.0001**
BMI, kg/m^2^	26.7 ± 4.0	26.4 ± 3.9	26.8 ± 4.0	**0.001**
Hypertension, i (%)	3,472 (70)	1,599 (75)	1,873 (67)	** <0.0001**
Dyslipidemia, *n* (%)	3,071 (62)	1,323 (62)	1,748 (63)	0.697
Diabetes mellitus, *n* (%)	1,310 (27)	640 (30)	670 (24)	** <0.0001**
Smokers, *n* (%)	1,193 (24)	445 (21)	748 (27)	** <0.0001**
Previous AMI, *n* (%)	1,671 (34)	819 (38)	852 (30)	** <0.0001**
LVEF, %	53.9 ± 11.5	52.5 ± 12.0	55.1 ± 10.9	** <0.0001**
eGFR, mL/min/1.73 m^2^	74.0 ± 24.8	69.8 ± 24.6	77.2 ± 24.4	** <0.0001**
AMI, *n* (%)	2,167 (44)	1,038 (49)	1,129 (40)	** <0.0001**

*AMI, Acute myocardial infarction; AVSc, Aortic valve sclerosis; BMI, Body mass index; eGFR, Estimated glomerular filtration rate; LVEF, Left ventricular ejection fraction. p-value < 0.05 are reported in bold*.

In the overall population, AVSc was detected in 2,138 (43%) patients. As expected, patients with AVSc were older and showed a higher prevalence of cardiovascular risk factors and previous AMI. Moreover, patients with AVSc had significantly lower LVEF and eGFR compared to patients without AVSc.

**Figure 2:**
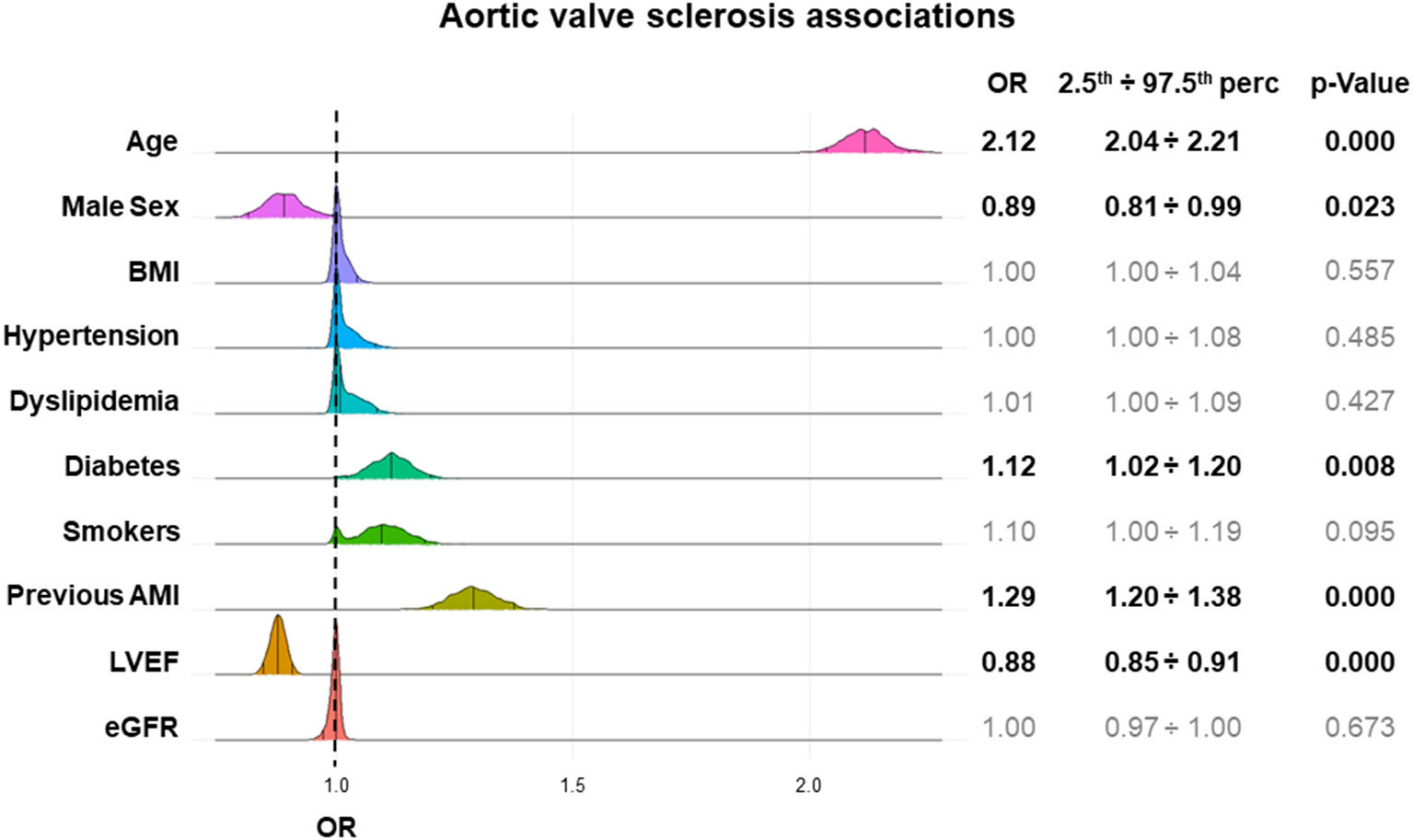
AVSc associations in high-risk CAD patients. The plot shows the strength of the associations between cardiovascular risk factors and AVSc (odds ratio–OR) and their dispersion. For each variable, the results obtained from multivariable logistic LASSO regressions after 1,000 bootstrap iterations are depicted and the OR (median, 2.5th percentile, and 97.5th percentile) and significance level are reported. The y-axis represents the frequency of a specific OR, while the x-axis represents the OR estimated values. AMI, acute myocardial infarction; BMI, body mass index; CAD, coronary artery disease; eGFR, estimated glomerular filtration rate; LVEF, left ventricular ejection fraction.

Multivariable LASSO logistic regression showed that age, diabetes, and previous AMI were positively associated, while male sex and LVEF were negatively associated with AVSc. The highest estimated odds ratios (OR) were related to age (2.12) and previous AMI (1.29), while the other variables had modest OR (Figure 2).

**Figure 3:**
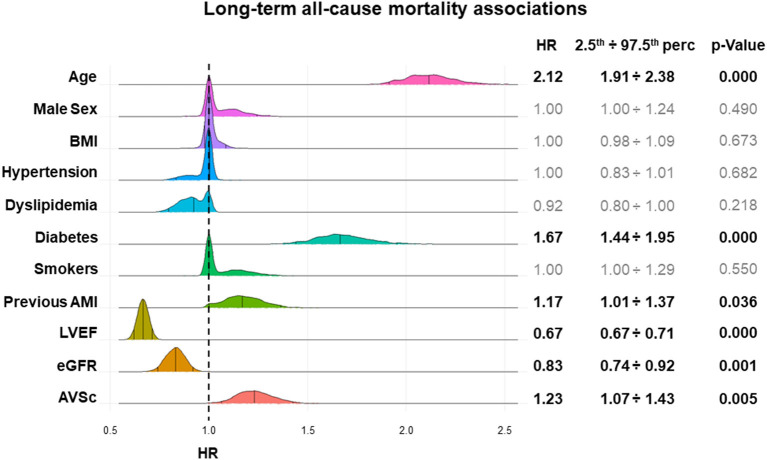
Variables associated with long-term all-cause mortality in high-risk CAD patients. The plot shows the strength of the associations between cardiovascular risk factors or AVSc and 5-year all-cause mortality (hazard ratio–HR) and their distribution. For each variable, the results obtained from multivariable Cox LASSO regressions after 1,000 bootstrap iterations are depicted and the HR (median, 2.5th percentile, and 97.5th percentile) and significance level are reported. The y- axis represents the frequency of a specific HR, while the x-axis represents the HR estimated values. AMI, acute myocardial infarction; AVSc, aortic valve sclerosis; BMI, body mass index; CAD, coronary artery disease; eGFR, estimated glomerular filtration rate; LVEF, left ventricular ejection fraction.

Over 5 years, we recorded 499 (11.5%) deaths. Patients with AVSc had a higher mortality rate than those without AVSc (16.6 vs. 7.4%; *p* < 0.0001). In the whole group, the multivariable LASSO regression analysis showed that AVSc was positively associated with all-cause mortality, along with age, diabetes, and previous AMI, whereas LVEF and eGFR were negatively associated. The strength of the association of AVSc with mortality was at least equal to that of previous AMI, and eGFR, which are well-established parameters associated with mortality (Figure 3).

**Figure 4:**
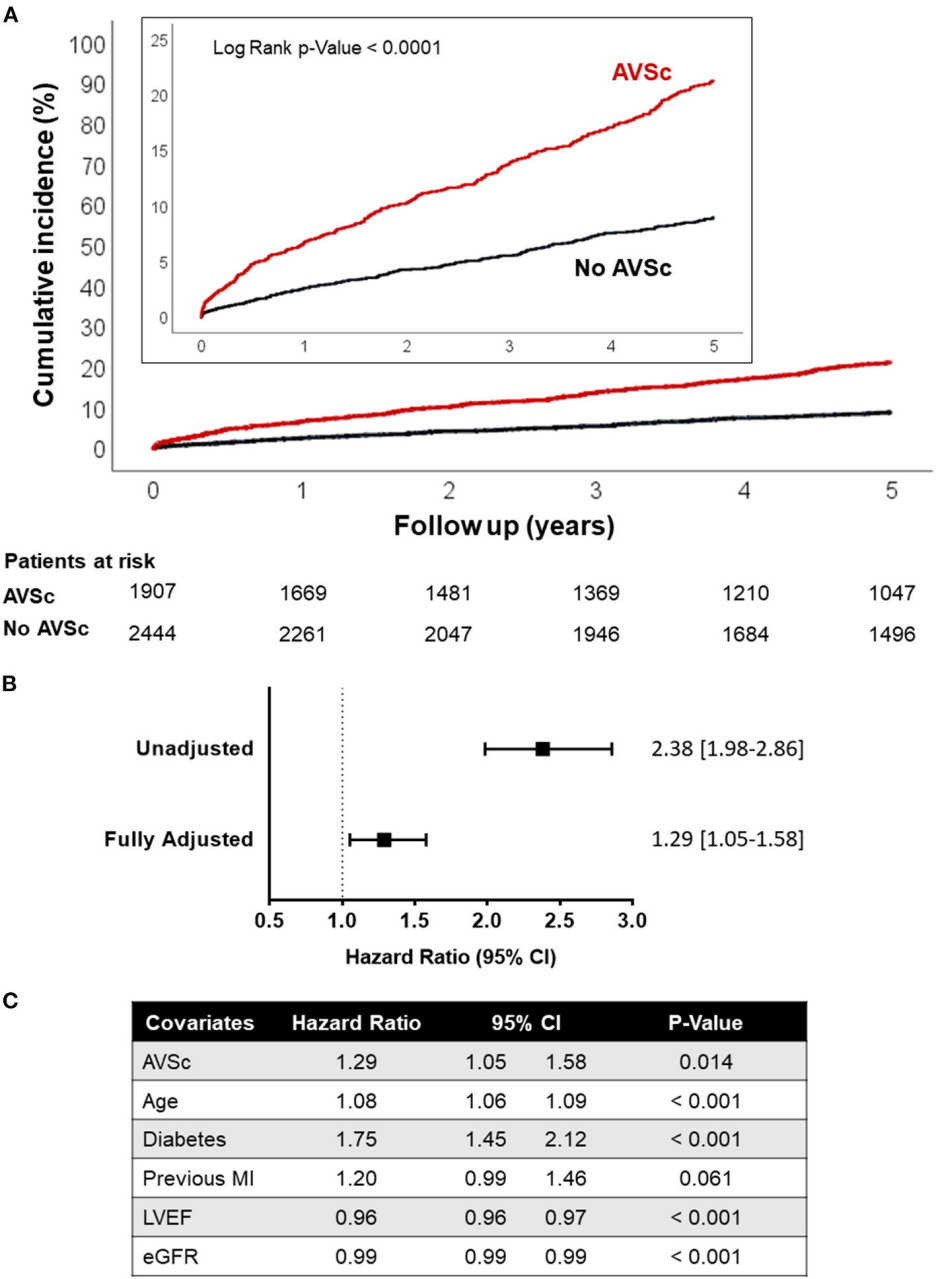
Long-term all-cause mortality in high-risk CAD patients. **(A)** Kaplan-Meier curves showing the cumulative incidence of all-cause mortality in high-risk CAD patients with AVSc vs. high-risk CAD patients without AVSc. **(B)** Cox-regression analyses showing the hazard ratios (HR) unadjusted and fully adjusted for age, diabetes mellitus, previous MI, LVEF, and eGFR. Square brackets indicate the 95% confidence interval. **(C)** Table showing the HR associated with all covariates considered in the fully adjusted model. AVSc, aortic valve sclerosis; CAD, coronary artery disease; eGFR, estimated glomerular filtration rate; LVEF, left ventricular ejection fraction; MI, myocardial infarction.

The Kaplan-Meier curves for 5-year all-cause mortality in patients with and without AVSc are shown in Figure 4A, while the unadjusted and adjusted hazard ratio (HR) for all-cause mortality associated with AVSc are shown in Figure 4B. The unadjusted Cox regression analysis revealed that AVSc patients had an increased risk of mortality at 5 years (HR 2.38, 95% CI 1.98–2.86; *p* < 0.001); this association withstood the adjustment (HR 1.29, 95% CI 1.05–1.58; *p* = 0.014) for all the confounders identified by the LASSO regression (i.e., age, diabetes mellitus, previous MI, LVEF, and eGFR; Figure 4C). To test whether AVSc could have an incremental predictive value over classical risk factors, we calculated both NRI and IDI for multivariate models predicting 5-years all-cause mortality with and without AVSc as a predictor. NRI showed that 13% of the cohort was better classified when AVSc was added to the prediction model (NRI: 0.13 [0.04–0.18]; *p* = 0.018); moreover, IDI shows a significant, albeit modest, improvement in prediction when AVSc was added to the model (IDI: 0.01 [0.003 – 0.02]; *p* < 0.001). Finally, we performed a subgroup analysis to evaluate whether the association between AVSc and 5-year all-cause mortality was similar between classes of age (below or above the median), sex, diabetes mellitus, dyslipidemia, hypertension, smoking habits, previous MI, LVEF, and eGFR (for both of the latter below or above the median). We found no significant difference in any of the subgroup comparisons (Supplementary Figure 1).

## Discussion

Our study shows that AVSc is detected in almost 50% of high-risk CAD patients and is associated with old age, female sex, diabetes, previous AMI, and low LVEF. In addition, high-risk CAD patients with signs of AVSc have about 30% increased risk of long-term mortality compared to high-risk CAD without AVSc.

In the last years, clinicians and scientists have increasingly studied AVSc, but it is still a matter of debate whether it is only a stage in the process of valve degeneration or a marker of systemic vascular disease (16). Aortic valve sclerosis, identified by echocardiography, is an abnormal non-uniform thickening of the aortic cusps (with or without calcium nodules) with preserved hemodynamics (2). In the general population, AVSc presence ranged between 9 and 42% of the subjects, depending on the average age of the study participants (16) and it has been shown that AVSc is strongly associated with CAD (5). However, no large study has been performed to specifically evaluate, in high-risk CAD patients, (i) AVSc prevalence, (ii) which cardiovascular risk factors are associated with AVSc, and (iii) whether AVSc is associated with long-term mortality.

Several studies reported the AVSc prevalence in CAD patients, showing a percentage ranging from 29 to 46% (6, 7, 17–19). However, only two studies (for a total of 540 enrolled patients) were focused on high-risk CAD patients (i.e., AMI) with a mean weighted AVSc prevalence of 44% (6, 17). In our cohort of almost 5,000 high-risk CAD patients, we have found that AVSc is present in 43% of cases. Thus, we confirmed that almost half of the high-risk CAD patients have an abnormal aortic valve with preserved hemodynamic. Regarding the cardiovascular risk factors associated with AVSc, besides age, a recent study performed on 368 AMI patients revealed that only previous AMI was associated with AVSc (6). Another study focusing on 1,024 AMI patients showed that current smoking was associated with AVSc (19). In contrast, in our cohort, we showed that also female sex, diabetes, and reduced cardiac function were associated with AVSc presence. Thus, our study revealed new and at least in part modifiable factors associated with AVSc in high-risk CAD patients.

The association between AVSc and long-term mortality risk has been investigated since 1999 with the first study performed by Otto et al. (2) on more than 5,500 community-dwelling adults with a mean age of 73 years. The authors highlighted that AVSc presence was associated with a 35% increase in the risk of all-cause mortality. In 2006, a second study on more than 3,500 community-dwelling adults with a mean age of 76 years, found similar results with a 23% increase in the risk of all-cause mortality in AVSc patients (20). In contrast, a successive study, evaluating more than 8,500 community-dwelling adults with a mean age of 56 years, showed that there was no association between AVSc presence and all-cause mortality (21). However, the last study, even if performed on a larger cohort, had an overall mortality of 3.5%, while in the two previous ones it was around 15%. This difference could be explained by the different ages of participants and the number of recorded events could have influenced the assessment in the multivariable-adjusted models. On the other hand, when the authors of the previous two studies focused their attention on CAD patients, they found that there was no association between AVSc and all-cause mortality in CAD subgroups (2, 20). Besides, a study focusing on patients with known CAD (n = 814), with a mean age of 66 years, reported no association between AVSc and all-cause mortality (19). Thus, it has been proposed that AVSc may potentially serve as a cardiovascular risk marker in patients without overt atherosclerosis (1). However, all the above-mentioned studies had a limited number of CAD patients and the true prognostic relevance of AVSc in CAD patients was not fully elucidated, especially in high-risk subjects. Our study, leveraging a large (*n* = 4,351) high-risk CAD population with a mean age of 67 years, allowed us to unveil that AVSc is independently associated with a 29% increased risk of all-cause mortality, even after adjusting for major confounders.

The mechanisms underlying the association between AVSc and adverse outcomes are still to be identified. Indeed, epidemiologic studies provide no insight into the biological processes of these associations. The progression of AVSc could lead to increased leaflet stiffness favoring calcium deposition with subsequent outflow obstruction (i.e., aortic stenosis) (22). Besides, several studies also support the hypothesis that endothelial dysfunction could be a factor of adverse outcomes in patients with AVSc since reduced brachial artery flow-mediated dilation and impairment of the glutathione system have been observed in these patients (23, 24). Chandra et al. (18) suggested that AVSc and atherosclerosis are both the result of increased systemic inflammation. However, it is not unequivocally proven that inflammation is the cause of adverse events in AVSc patients yet (25) and this issue warrants further investigation. Hence, it is still unclear if the biological processes linking AVSc to adverse clinical events are diffuse atherosclerosis, altered calcium metabolism, endothelial dysfunction, lipid accumulation and oxidation, genetic variants, or other unknown factors (9, 25).

That being said, the detection of AVSc could help physicians to early recognize, among high-risk CAD patients, those who can derive the greatest benefit from a closer clinical follow-up, a more intensive therapy, and a more rigorous risk factor control. Indeed, timely decision-making is especially desirable for the prevention of further fatal and non-fatal ischemic events, considering that the first years after AMI or CABG surgery are those at the highest risk of recurrence (26, 27). Moreover, the ability to early identify CAD patients who are at high risk may be useful for testing novel pharmacological and non-pharmacological preventive therapies. Nevertheless, future studies are warranted to investigate whether the implementation of stringent secondary prevention strategies to high-risk CAD patients with AVSc may improve their outcome.

### Limitations

The strengths of our study are represented by a large, well-characterized population of high-risk CAD patients, in whom the prognosis is mainly driven by their CAD, and a special focus on AVSc detection. However, there are important considerations pertinent to the methods of this study. First, all the patients were recruited retrospectively, acquiring demographic, clinical, and only a few echocardiographic data, at a single center specialized in cardiovascular disease. Nonetheless, the high volume of echocardiography performed allows us an accurate reporting of aortic valve appearance. Second, the outcome evaluated in our study is all-cause mortality since we do not have access to re-hospitalization as well as cardiovascular mortality data but we have to consider that 70–80% of long-term deaths, experienced by high-risk CAD patients, are cardiovascular in nature (25, 28). Third, we have to consider that 30% of patients with AVSc develop some degree of aortic stenosis over an average period of 4 years (29) and this might have influenced the outcome of our study. Fourth, the lack of association between AVSc and dyslipidemia, which should be expected in light of recent findings (30), might be explained by the fact that we retrieved the dyslipidemia status from anamnestic records, not having the actual lipid profile of our patients. Finally, our study included only high-risk CAD patients and whether our results apply also to all CAD patients cannot be inferred from our data.

## Conclusions

The AVSc is frequently detected in high-risk CAD patients and is associated with long-term mortality. Our findings corroborate the hypothesis that AVSc is an underestimated indicator of systemic cardiovascular damage that represents a potential red flag of a worst cardiovascular condition associated with high mortality risk in CAD patients. Thus, AVSc detection may be used to improve long-term risk stratification of high-risk CAD patients.

## Data Availability Statement

The raw data supporting the conclusions of this article will be made available by the authors, without undue reservation.

## Ethics Statement

The studies involving human participants were reviewed and approved by the Institutional Review Board and by the Ethical Committee of CCM (R553/17-CCM 591). The patients/participants provided their written informed consent to participate in this study.

## Author Contributions

VM: conception of the study, data collection, and writing the manuscript. GM and SG: conception of the study and critical review of the manuscript. NC and JC: data collection and critical review of the manuscript. SG, LC, JC, MR, DB, MP, FA, and GC: critical review of the manuscript. AB, MC, and FV: data analysis and critical review of the manuscript. PP: conception of the study, data collection, data analysis, and writing the manuscript. VM and PP were responsible for the overall content as guarantors. All authors contributed to the article and approved the submitted version.

## Conflict of Interest

The authors declare that the research was conducted in the absence of any commercial or financial relationships that could be construed as a potential conflict of interest.

## Publisher's Note

All claims expressed in this article are solely those of the authors and do not necessarily represent those of their affiliated organizations, or those of the publisher, the editors and the reviewers. Any product that may be evaluated in this article, or claim that may be made by its manufacturer, is not guaranteed or endorsed by the publisher.

## Abbreviations

AMI: acute myocardial infarction
AVSc: aortic valve sclerosis
CABG: coronary artery bypass grafting
CAD: coronary artery disease
eGFR: estimated glomerular filtration rate
HR: hazard ratio
LASSO: least absolute shrinkage and selection operator
LVEF: left ventricular ejection fraction
OR: odds ratio.
