# Supplementary material for: Aortic Valve Sclerosis in High-Risk Coronary Artery Disease Patients
Source: Front Cardiovasc Med. 2021 Jul 27;8:711899. doi: 10.3389/fcvm.2021.711899 (PMC8354333; doi:10.3389/fcvm.2021.711899)
Supplement: Supplementary file 1 [file Data_Sheet_1.docx]

**Supplementary Files**

**Aortic Valve Sclerosis in High-Risk Coronary Artery Disease Patients**

*Veronika A. Myasoedova^1^MD, PhD, Stefano Genovese^1^ MD, PhD, Laura Cavallotti ^1^ MD, PhD, Alice Bonomi^1^ PhD, Mattia Chiesa^1^PhD, Jeness Campodonico^1^MD, Maurizio Rondinelli^1^MD, Nicola Cosentino^1^MD, Damiano Baldassarre^1^PhD, Fabrizio Veglia^1^PhD, Mauro Pepi^1^MD, Francesco Alamanni^1^MD, Gualtiero I. Colombo^1^MD, PhD, Giancarlo Marenzi^1^MD,*

*and Paolo Poggio^1,#^PhD*

^1^Centro Cardiologico Monzino IRCCS, Milan, Italy.

**Condensed title:** *AVSc and high-risk CAD patients.*

^#^To whom correspondence should be addressed:

Paolo Poggio, PhD

Unit for the Study of Aortic, Valvular and Coronary Pathologies

Centro Cardiologico Monzino IRCCS

Via Carlo Parea 4, 20138 Milan, Italy

Telephone: +39 02.5800.2853

Fax: +39 02.5800.2750

Email: [paolo.poggio@ccfm.it](mailto:paolo.poggio@ccfm.it)

**Supplementary Table S1.** Demographic and clinical characteristics of the high-risk coronary artery disease patients analysed in the primary objective versus patients lost at follow up.

| **Variables** | **Patients analysed for Primary Objective**  (n = 4938) | **Patients lost at Follow Up**  (n = 587) | **p-Value** |
| --- | --- | --- | --- |
| Age, years | 66.8 ± 10.7 | 65.8 ± 9.7 | **0.012** |
| Male n, (%) | 3954 (80) | 500 (85) | **0.003** |
| BMI, kg/m^2^ | 26.7 ± 4.0 | 27.1 ± 4.1 | **0.008** |
| Hypertension, n (%) | 3472 (70) | 444 (76) | **0.007** |
| Dyslipidemia, n (%) | 3071 (62) | 405 (69) | **0.001** |
| Diabetes mellitus, n (%) | 1310 (27) | 187 (32) | **0.006** |
| Smokers, n (%) | 1193 (24) | 110 (19) | **0.003** |
| Previous AMI, n (%) | 1671 (34) | 216 (37) | 0.155 |
| LVEF, % | 53.9 ± 11.5 | 55.6 ± 11.0 | **0.002** |
| eGFR, mL/min/1.73m^2^ | 74.0 ± 24.8 | 73.6 ± 24.4 | 0.686 |
| AMI, n (%) | 2167 (44) | 111 (19) | **< 0.0001** |

AMI: Acute myocardial infarction; AVSc: Aortic valve sclerosis; BMI: Body mass index; eGFR: Estimated glomerular filtration rate; LVEF: Left ventricular ejection fraction.

**Supplementary Figure S1. Forest plot of subgroup analysis.** Association between AVSc and 5-year all-cause mortality evaluated within subgroups and expressed as a hazard ratio with 95% confidence interval and the respective interaction.
