# Supplementary material for: Aortic Valve Sclerosis in High-Risk Coronary Artery Disease Patients
Source: Front Cardiovasc Med. 2021 Jul 27;8:711899. doi: 10.3389/fcvm.2021.711899 (PMC8354333; doi:10.3389/fcvm.2021.711899)
Supplement: Supplementary file 2 [file Presentation_1.PPTX]

## Slide 1
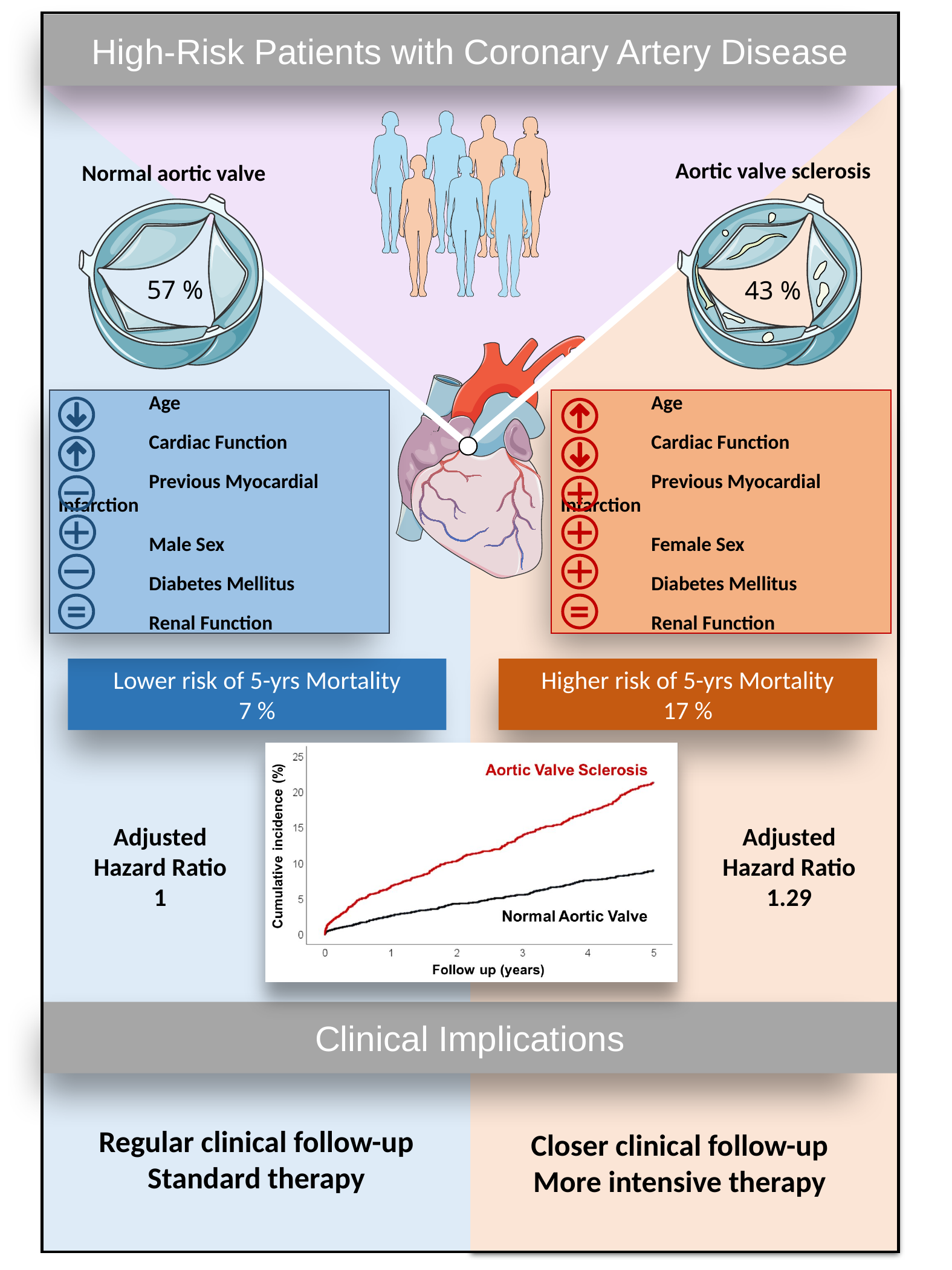

High-Risk Patients with Coronary Artery Disease
Aortic valve sclerosis
Normal aortic valve
57 %
43 %
	Age
	Cardiac Function
	Previous Myocardial Infarction
	Male Sex
	Diabetes Mellitus
	Renal Function
	Age
	Cardiac Function
	Previous Myocardial Infarction
	Female Sex
	Diabetes Mellitus
	Renal Function
=
=
Lower risk of 5-yrs Mortality
7 %
Higher risk of 5-yrs Mortality
17 %
Adjusted Hazard Ratio
1
Adjusted Hazard Ratio
1.29
Clinical Implications
Regular clinical follow-up
Standard therapy
Closer clinical follow-up
More intensive therapy
